# Role of *IGF2* in the Study of Development and Evolution of Prostate Cancer

**DOI:** 10.3389/fgene.2021.740641

**Published:** 2022-01-04

**Authors:** P. Porras-Quesada, JM. González-Cabezuelo, V. Sánchez-Conde, I. Puche-Sanz, V. Arenas-Rodríguez, C. García-López, JF. Flores-Martín, JM. Molina-Hernández, MJ. Álvarez-Cubero, LJ. Martínez-González, F. Vázquez-Alonso

**Affiliations:** ^1^ Centre for Genomics and Oncological Research: Pfizer, University of Granada, Andalusian Regional Government (GENYO), Granada, Spain; ^2^ Research and Development Department, Meridiem Seeds, Almería, Spain; ^3^ Urology Department, University Hospital Virgen de las Nieves, Granada, Spain; ^4^ Pathological Anatomy Service, University Hospital Virgen de las Nieves, Granada, Spain; ^5^ Urology Department, University Hospital of Jaen, Jaen, Spain; ^6^ Urology Department, University Hospital Torrecárdenas, Almería, Spain; ^7^ Department of Biochemistry and Molecular Biology III, Faculty of Medicine, University of Granada, Granada, Spain; ^8^ Biosanitary Research Institute (ibs. GRANADA), University of Granada, Granada, Spain

**Keywords:** biomarker, expression patterns, *IGF2*, miRNA, miR-93-5p, precision medicine, prostate cancer

## Abstract

Prostate Cancer (PC) is commonly known as one of the most frequent tumors among males. A significant problem of this tumor is that in early stages most of the cases course as indolent forms, so an active surveillance will anticipate the appearance of aggressive stages. One of the main strategies in medical and biomedical research is to find non-invasive biomarkers for improving monitoring and performing a more precise follow-up of diseases like PC. Here we report the relevant role of *IGF2* and miR-93-5p as non-invasive biomarker for PC. This event could improve current medical strategies in PC.

## Introduction

It is well known that prostate cancer (PC) is a heterogeneous disease, which makes it difficult the identification of any clinical and molecular biomarker in disease management. PC is one of the most frequently diagnosed tumors among men in Europe and reaches the second position when comparing data worldwide, with over 1.4 million diagnoses recorded in 2020 (WHO, 2020). The use of robust biomarkers; mainly focused on molecular non-invasive ones; is still a challenge in this tumor. Several germline variants have been suggested as relevant in PC such as those in *ATM* (ataxia-telangiectasia mutated), *BRCA1* (breast cancer), *BRCA2, MSH2* (MutS Homolog 2)*, MLH1* (mutL homolog 1), *MSH6* (MutS Homolog 6), *PMS2* (PMS1 homolog 2), *EPCAM* (epithelial cellular adhesion molecule) and *HOXB13* (Homeobox B13) genes (Saunders et al., 2021). Additionally, recent data support the role of several SNPs in *IL-6* (Interleukin 6) gene (rs1800795, rs1800796 and rs1800797) as biomarkers of an increased cancer risk in several tumors. Specially, variants rs1800795 and rs1800796 are associated with an overall increased risk of PC (Harun-Or-Roshid et al., 2021).

**GRAPHICAL ABSTRACT:**
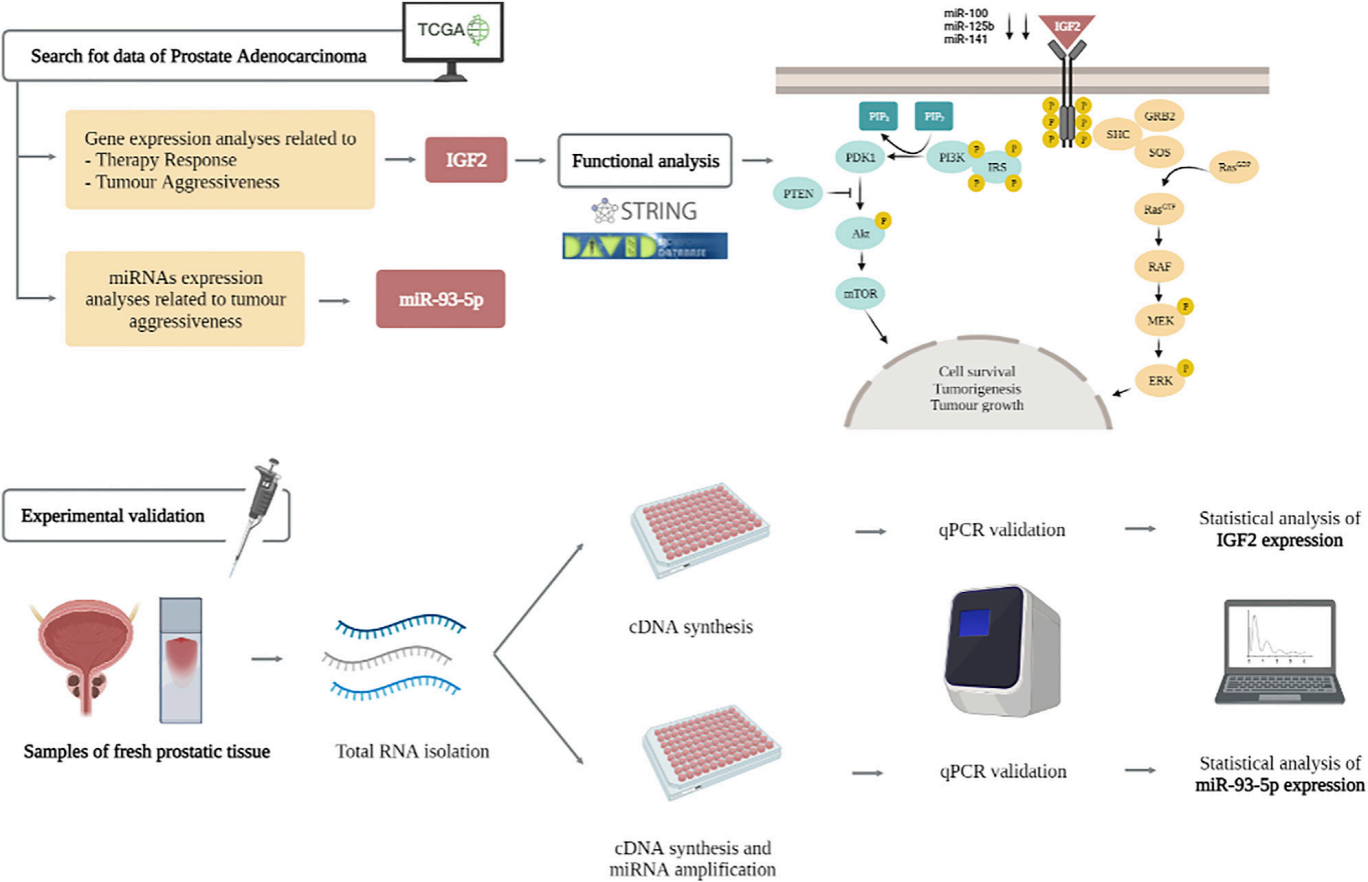


Here, we focus on the role of *IGF2* (insulin-like growth factor 2) as a novel marker for PC management. *IGF2* encodes a member of the insulin family of polypeptide growth factors, which are involved in development, cancer biology and growth. It binds to type-1 insulin-like growth factor receptor (*IGF1R*), which activates downstream members of the *PI3K* (phosphatidylinositol 3-kinase)/*AKT* (alpha serine/threonine-protein kinase) and *MAPK* (mitogen-activated protein kinases)/*ERK* (extracellular signal-regulated kinases) pathways. This gene is important for cell survival and tumorigenesis; moreover it has been suggested that *IGF2*, in combination with *SSTR2* (Somatostatin Receptor 2), plays an important role in PC survival. Previous data have focused on the role of *IGF2* expression patterns or epigenetic imprinting in PC (Cao et al., 2014; GeneCard, 2021); and other tumors such as colon cancer, detecting recurrent fusions in this gene (Yun et al., 2020). Furthermore, *IGF2* messenger RNA binding protein 3 (*IMP3*) has been reported to be over-expressed in PC and strongly correlated to poor prognosis. The main role of *IMP3* (U3 small nucleolar ribonucleoprotein) has been included in PI3K/AKT/mTOR signalling pathway (Zhang et al., 2020). The relevance of *IGF2* in PC has been denoted not only in mRNA, but also in lncRNA and SNPs. That is the case of *IGF2AS* (*IGF2* Antisense RNA), which has been proposed as an epigenetic tumor suppressor in human PC. Moreover, the relationship between *IGF2AS/*and *IGF2* has been included as a possible marker for future therapeutic targets in PC treatment or gastric cancer (Chen et al., 2019; Xing et al., 2021). Both, *IGF2* and its receptor *IGF1R* constitute desirable therapeutic targets; mainly due to these evidences showing that targeting either *IGF2* or its receptor *IGF1R*, blocks cancer progression and displays significant antitumor activity (Xing et al., 2021).

There are several SNPs in *IGF2* that have been previously reported to have a role in cancer or other diseases; such as rs1004446. This SNP has been previously proposed as a marker of decreased endometrial cancer risk (McGrath et al., 2011); or increased PC risk (Cao et al., 2014). Another *IGF2* SNP, rs4320932, has been associated with a decreased risk of ovarian cancer in G allele carriers (Pearce et al., 2011).

Several miRNAs have been also evaluated with a relevant role in *IGF2* regulation. That is the case of miR-100 and miR-125b, which play a proven tumor suppressor role in hepatocellular carcinoma, by inhibiting *IGF2* expression and activating *AKT/mTOR* pathway (Seol et al., 2020). miR-141 down-regulation blocks *VEGF* (Vascular Endothelial Growth Factor) and *IGF2* expression; and also interacts with osteoblasts proliferation, which is relevant in osteosarcoma (He et al., 2016). In pancreatic cancer, it has also been proven the role of miR-141 in *IGF2BP2* (*IGF2* mRNA-binding protein 2); which is known to play oncogenic roles. Genomic amplification and silencing of miR-141 also contribute to *IGF2BP2* activation; opening promise molecular targets in pancreatic tumors (Xu et al., 2019).

Concerning to somatic mutations, there is scarce data in PC, mainly due to current methodological strategies limitations in their analysis of because of their location in non-coding regions. In PC, their effects on driving tumorigenesis and progression have not been systematically explored (Wang and Li, 2021). We have previously published the role of several somatic mutations, just discovering an incipient role of c.1621A > C (rs3822214) in *KIT* (tyrosine kinase), c.38G > C (rs112445441) in *KRAS* (kirsten rat sarcoma virus) and c.733G > A (rs28934575) in *TP53* (tumor protein) genes among patients with PC; although with weak associations (Martinez-Gonzalez et al., 2018). Others authors suggested the association of increased expression patterns of homeobox B13 (*HOXB13*), a gene related to normal prostate development, with worse outcomes after PC surgery (Weiner et al., 2020).

Here, we perform an integrated analysis combining bioinformatic and experimental analyses proving the role of *IGF2* as a marker in PC. We focus on two main SNPs and miRNAs interactions in PC. The use of both biomarkers (SNPs and miRNAs) could be easily developed in clinical routine practice, mainly by the low cost of these methodologies.

## Materials and Methods

### Study Population for Experimental Analysis

Present study includes data from 199 men with prostate specific antigen (PSA) values above 4 ng/ml and histological confirmed PC. A total of 30 EDTA (ethylenediamine tetraacetic acid) blood samples and 38 buccal swabs from these subjects were collected by the Urology Service from “Hospital Universitario Virgen de las Nieves de Granada, Spain.” Samples were stored at −20°C until they were processed. Both, blood samples and buccal swabs, were used for SNPs genotyping analysis.

For RNA expression analysis, 131 fresh tissue samples were collected from nearly 66% of the patients of present study; these samples were stored at −80°C until they were processed. Concerning mRNA analysis, just 78 samples (39.2% of the total samples) were available (mainly limited by the quality of mRNA). Moreover, for miRNAs analysis we included all 131 fresh tissue samples of PC patients and 28 controls. Several clinical data of samples were collected such as age, Gleason Score, minimum PSA value, and treatment follow-up (Table 1). All study participants provided a written informed consent before being enrolled, and the study was previously approved by the Research Ethics Committee of Granada Center (CEI-Granada internal code 1638-N-18) following Helsinki ethical declaration. A supplementary figure explaining this sample distribution is included in Supplementary Figure S1.

**TABLE 1:** Descriptive variables of PC samples.

Variables	PC n* (%)
Age (years)	
<60	5 (7.69%)
60–69	19 (29.23%)
70–79	30 (46.16)
≥80	11 (16.92%)
PSA level (ng/ml)	
<20	37 (47.44%)
≥20	41 (52.56%)
Gleason score	
≤7	44 (53.01%)
>7	39 (46.99%)
D’Amico Risk Classification	
Low	8 (10.53%)
Medium	20 (26.31%)
High	48 (63.16%)
Treatment Response	
Sensitivity	35 (38.04%)
Resistance	57 (61.96%)
Metastasis	
Yes	45 (48.91%)
No	47 (51.09%)

n*(some reports data are missing; for that reason, the total number of samples do not sum the same total).

### Bioinformatic Analysis

This analysis was performed by the access to “The Cancer Genome Atlas (TCGA)” which was initiated in 2005 and, as of today, it has over 2.5 petabytes data of 20,000 primary cancers and matched normal samples from 33 cancers types. TCGA was created as an easy way of exploring the entire spectrum of genomic changes involved in human cancer (Cancer Genome Atlas, 2021). This makes TCGA repositories an extraordinary-value source of data in studies like the present one.

#### TCGA Data of Prostate Adenocarcinoma

From the Broad Institute GDAC (Genome Data Analysis Center) (Broad Institue, 2021), we extracted all available Gene expression (mRNA-Seq) data of PRAD (a total of 550 cases), containing both tumoral (T) and non-tumoral (NT) tissue samples at preprocess level [prostate PRAD (cancer type), RNASeqV2 (data type), level 3 (archive type) and 2016-02-13 (data version)]. Data were generated based on Illumina HiSeq 2000 platform and annotated to reference transcript set of UCSC hg19 gene standard track. One single sample was removed from the set to prevent possible disturbances in the results as it corresponded to a metastatic sample. In addition, a total of 531 Isoform Expression Quantification (miRNA-Seq) files containing both tumoral (T) and non-tumoral (NT) tissue samples, as well as clinical data for each patient/sample was obtained from TCGA data portal (NIH, 2021a). All data are controlled; the access has been requested through the GDAC of the National Institutes of Health (NIH).

#### TCGA Differential Expression Analyses

Differential expression analyses have been carried out using edgeR (version 3.28.0) Bioconductor package (Robinson et al., 2009; McCarthy et al., 2012). Quasi-likelihood F-test (QL) from the GLM (Generalized Linear Models) was used to determine differentially expressed genes (DEG) related to tumor aggressiveness and treatment effectiveness. For that purpose, two different analyses were performed, in which different groups were established based on the clinical information of each case:

1) DEG related to AD (androgen deprivation) therapy response: Three categories were established [DR1: treatment based on a single drug target (43 cases)], DR2: treatment in which a new drug target has been prescribed due to failure of the first one (25 cases), DR3: chemotherapy (5 cases). Four drug targets apart from the chemotherapy were considered: LHRH agonists, LHRH antagonists, antiandrogens, and CYP17 inhibitors. NT (non tumor) samples, as well as those lacking information on treatment, were excluded from this analysis.
2) DEG related to Gleason score: Three categories were defined [G0 = NT samples (52 cases), G1 = Gleason score equal or lower than 7 (292 cases), G2 = Gleason score higher than 7 (205 cases)].

To determine differentially expressed (DE) miRNA related to tumor aggressiveness, three categories were defined [G0 = NT samples (51 cases), G1 = Gleason score equal or lower than 7 (285 cases), G2 = Gleason score higher than 7 (195 cases)]. As for mRNA-Seq, Quasi-likelihood F-test (QL) from the GLM (Generalized Linear Models) was used to perform the analysis.

Low expression filter was applied for every single analysis by using as representative threshold the number of samples of the group that has less expression values. The normalization of the samples was calculated using the “*calcNormFactors*” function and the trimmed mean of M-values (TMM) method (Robinson and Oshlack, 2010), while the dispersions were estimated using the “*estimateGLMCommonDisp*,” “*estimateGLMTagwiseDisp*,” and “*estimateGLMTrendedDisp*” functions. Three contrasts were carried out in each analysis:

- DR2 vs. DR1, DR3 vs. DR1, and DR3 vs. DR2 for AD therapy response (mRNA-Seq).
- G1 vs. G0, G2 vs. G0, and G2 vs. G1 for Gleason score (mRNA-Seq).
- G1 vs. G0, G2 vs. G0, and G2 vs. G1 for Gleason score (miRNA-Seq).

In all our DE analyses, the *p* value was adjusted by Benjamini–Hochberg false discovery rate (FDR) procedure (Benjamini et al., 2001).

We used STRING (Search Tool for the Retrieval of Interacting Genes/Proteins) database (Szklarczyk et al., 2019) to select DEGs along with *IGF2*. To do so, we performed the *IGF2* interactor search according to the default parameters within “single protein by name” option and selecting no more than 20 interactors in first shell. From all of them, we choose those that exceeded a score of 0.980. Additionally, *IGF2* interactors with particular significance in PC such as *VEGFA, STAT3, FLT1, KDR, NRP1, NRP2*, and *HIF1A* were manually added to complete 20 interactors.

On the other hand, the selection of miRNAs of interest was carried out using MirTarBase (miRTarBase, 2021) through the search of *IGF2* by target gene according to default parameters.

#### TCGA Somatic Mutations

SNVs (single nucleotide variants)/mutations may affect gene function occasionally leading to a total loss of function (LOF). This is related to the development and prognosis of a tumor. For this reason, we study the alterations at the mutation level of *IGF2* gene in TCGA-PRAD cohort. To this end, we obtained 503 annotated somatic mutation files (MuTect2 annotation type) corresponding to TCGA-PRAD from TCGA data portal (NIH, 2021a). First, each of the Variant Call Format (VCF) file was parsed to extract information regarding the mutated genes presented in each sample. In a subsequent step, the result of each VCF file was checked against *IGF2* gene.

### *In Silico* Analysis

Based on data available in Genome Browser of UCSC (University of California, Santa Cruz), we obtained a total of 60 different SNPs of *IGF2* gene (Kent, 2002). An analysis of these *IGF2* variants was carried out in “The variant effect predictor” (McLaren et al., 2016). This software was used to calculate changes in transcripts and malignancy of variants. We also used ClinVar tool (Landrum, 2018) for data validation (only 6 of the 60 SNPs were available in this software). These analyses were performed to confirm the interactions in this gene with other germline or somatic variants, as well as miRNAs. Main results are included in Supplementary Table S1.

### Functional Analysis

Pathway analysis in *IGF2* gene was evaluated using DAVID (Database for Annotation, Visualization and Integrated Discovery) Bioinformatics Resources v6.8 (https://david.ncifcrf.gov/, accessed on November 1, 2021) to obtain the role of gene pool, clinical implication, ontology and involved metabolic pathways. Moreover, STRING search tool was used to calculate Interacting Genes with our target and interaction among our target genes (https://string-db.org/, accessed on November 1, 2021) (Szklarczyk et al., 2019).

### Molecular Analyses

This section pretends to validate main results obtained by bioinformatic analysis. We performed several analytic procedures contains SNPs genotyping and mRNA and miRNA expression analysis.

#### SNPs Selection and Genotyping

Out of the *IGF2* gene SNVs annotated with clinical association in public databases such as *The National Center for Biotechnology Information* (NCBI, 2019), we selected two (rs1004446 and rs3741211) for the present study. Only those SNVs with an allele frequency higher than 20% on the minor allele (MAF) in the Caucasian population according to the Ensembl database (Ensembl, 2021) were taken into consideration, more details of the probes can be found in Supplementary Table S2.

Each buccal swab or blood sample DNA was extracted using organic extraction reagents (1 ml de Stain Extraction Buffer + Proteinase K). DNA extraction protocol was performed as described by Freeman et al. (2003) and optimizations developed by Gomez-Martín A. et al. (Gómez-Martín et al., 2015). All samples were standardized to 20 ng/μl using Nanodrop 2000/2000c (ThermoFisher, United States) quantification. DNA genotyping was performed using TaqMan® Genotyping Master Mix (Applied Biosystems, United States) which included all essential components (except probes, templates and water) for polymerase chain reaction (PCR). Allelic discrimination assays were carried out in a 7900HT Fast Real-Time PCR System (Applied Biosystems, United States). Results were analyzed using SDS software v.2.4 (Applied Biosystems, United States).

#### mRNA Analysis

mRNA from a total of 78 fresh tissue samples was extracted using Trizol®/chloroform method and quality validated by A260/A280 in NanoDrop™ 2000c. Only those samples with the best quality and including all clinical records were selected. Reverse transcription was performed with TaqMan™ Advanced mRNA cDNA Synthesis kit (Applied Biosystem, Foster City, CA). Quantitative polymerase chain reaction (qPCR) was performed with SYBR Green designed probes (Life Technologies, Carlsbad, CA), on a 96-wells plate with QuantStudio 6 Flex Real-Time PCR System (Applied Biosystems). qPCR reactions were performed as follows: 95°C during 10 min for enzyme activation; followed by 40 cycles of 15 s at 95°C and 1 min at 60°C for denaturing and annealing/extension. Primers were designed using *Primer-Blast* (Ye et al., 2012) (*NIH*) *software* under the following conditions: they must span an exon-exon junction, have a PCR product size between 60–150 nucleotides and have a primer melting temperature within the range of 59–61°C. *Sigma Aldrich* company designed the primers with the following sequences UG_GX_IGF2_f (Forward): CGC​TGT​TCG​GTT​TGC​GAC, and UG_GX_IGF2_r (Reverse): GGA​TTC​CCA​TTG​GTG​TCT​GGA.

All samples were run in triplicates, with a NTC (non template control) in each plate. Threshold cycles (C_T_) ≥ 35 were considered as undetermined values. mRNAs expression levels were quantified using the comparative threshold cycle (Ct) method (2^−ΔΔCt^) relative to *HPRT1* (hypoxanthine phosphoribosyltransferase 1) expression as an endogenous control. Firstly, difference between *IGF2* and *HPRT1* expression was calculated for each sample (ΔC_T_ = C_T__IGF2_^−^ C_T__HPRT1_). Normalization was done using the mean of reference group; treatment sensitivity group for therapy response analysis; and Gleason ≤7 group for aggressiveness study (ΔΔC_T_ = ΔC_T_^−^ ΔC_T__reference_). Relative quantification parameter (RQ or 2^−ΔΔCt^) was estimated for each case and used in statistical analysis.

#### miRNA Analysis

Total RNA of 159 fresh tissue biopsies (including patients and controls) were extracted using Trizol®/chloroform method and quality validated by A260/A280 in NanoDrop™ 2000c. Reverse transcription was performed with TaqMan™ Advanced miRNA cDNA Synthesis kit (Applied Biosystem, Foster City, CA). Quantitative polymerase chain reaction (qPCR) was performed with TaqMan™ probes (Life Technologies, Carlsbad, CA), according to manufacturer’s protocol, on a 96-wells plate with QuantStudio 6 Flex Real-Time PCR System (Applied Biosystems). qPCR reactions were performed as follows: 5°C during 20 s for enzyme activation; followed by 40 cycles of 1 s at 95°C and 20 s at 60°C for denaturing and annealing/extension. For liquid biopsy analysis, plasma of 60 samples was isolated from blood; this process was carried out, at most, 4 h after collection. Total RNA of the samples was extracted using the miRNeasy Serum/Plasma Kit (Qiagen GE). All samples were run in triplicate, with a NTC in each plate.

We included the analysis of miR-93-5p, as one of the most interesting miRNAs according to bioinformatic analysis. miRNAs expression levels were quantified using the comparative Ct method (2^−ΔΔCt^) relative to *RNU6B* (U6B small nuclear RNA) expression as an endogenous control.

### Statistical Analysis

All analyses were performed using the SPSS v.22 statistical package (IBM Corporation, United States). Relationships between different genotypes and clinical variables were studied using the χ^2^ test. Odds Ratios (OR) and 95% confidence intervals (95% CI) were calculated by binary logistic regression. Genotypes analyses, Hardy–Weinberg equilibrium and Linkage disequilibrium (LD) analyses were performed using the online SNPStats software (Solé et al., 2006). SNPs are considered to be in LD when they have a value of r^2^ > 0.5. Present SNPs were in LD. For expression analysis, Shapiro-Wilks test was used to test the normalization of the samples. This test revealed that our results did not follow a gauss distribution, therefore a non-parametric test (U-Mann Whitney test) was performed for all variables. The level of statistical significance used was *p* < 0.05.

## Results

The main features of the study population stratified by PC and treatment response are shown in Table 1. First, we conducted a bioinformatic analysis and most relevant data, such as rs1004446 (*IGF2*) and mir-93-5p, were selected for being 199 samples (more details in Supplementary Figure S1).

### Bioinformatic Analysis (TCGA)

#### Gene Expression Analysis

**FIGURE 1:**
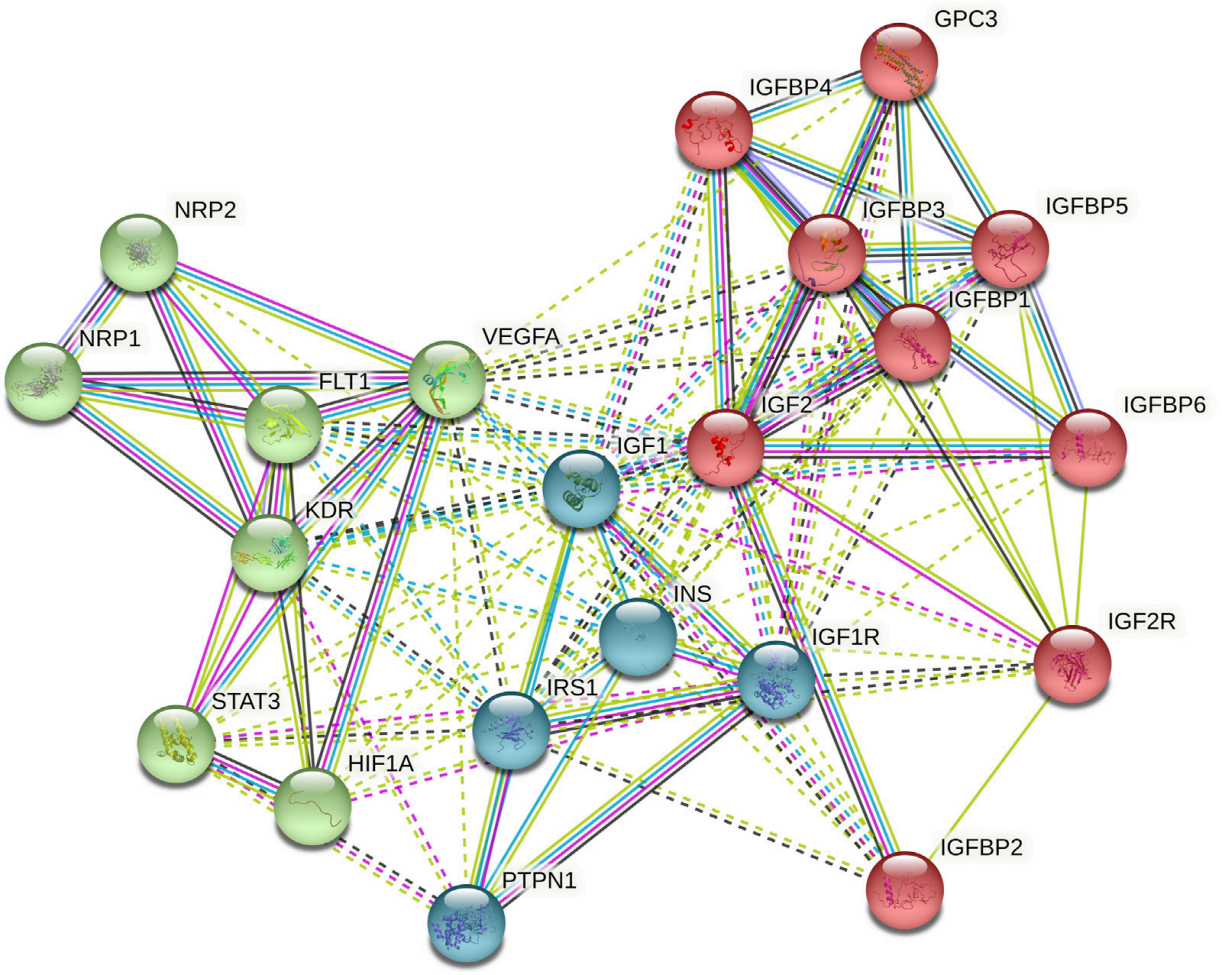
STRING network (default k-means clustering method) performed by the introduction of IGF2 with 20 interactors.

Out of all the DEGs obtained in differential expression analyses, we focused on *IGF2* and some of its closest interactors obtained by using the STRING database. The protein-protein interaction network obtained can be seen in Figure 1.

According to the obtained results by G0 vs. G1, G0 vs. G2, and G1 vs. G2, the most interesting genes in terms of *p*-values and FDR are *NRP2*, *KDR*, and *IGF2*. These results can be seen in Table 2. Data obtained from differential expression analysis based on AD therapy response are shown in Supplementary Table S3. Except for *IGF2*, which is under-expressed in patients with treatment resistance, any other gene showed any statistically significant result.

**TABLE 2:** Differential Expression of *IGF2* and *IGF2* interacting protein genes using Gleason score.

Gene	LogFC	FDR	*p* value	Test
*NRP2*	−1.3309	1.08E-18	4.55E-20	G0 vs. G1
*IGFBP2*	0.9156	2.74E-09	5.53E-10	G0 vs. G1
*KDR*	−0.8654	2.98E-09	6.02E-10	G0 vs. G1
*IGFBP3*	−0.6879	1.08E-06	3.27E-07	G0 vs. G1
*IGFBP4*	−0.5516	4.89E-05	1.95E-05	G0 vs. G1
*IGF2R*	0.4125	0.0002	9.81E-05	G0 vs. G1
***IGF2***	−**0.9725**	**0.0008**	**0.0004**	G0 vs. G1
*HIF1A*	−0.3241	0.0015	0.0008	G0 vs. G1
*IGFBP5*	−0.4570	0.0022	0.0012	G0 vs. G1
*IRS1*	−0.4073	0.0042	0.0023	G0 vs. G1
*VEGFA*	−0.5247	0.0145	0.0089	G0 vs. G1
*IGF1R*	0.2770	0.0411	0.0278	G0 vs. G1
*NRP2*	−1.0033	2.46E-10	4.61E-11	G0 vs. G2
*KDR*	−0.7398	1.28E-06	4.17E-07	G0 vs. G2
*IGFBP4*	−0.6542	2.88E-06	9.91E-07	G0 vs. G2
*IGFBP2*	0.6512	2.29E-05	9.07E-06	G0 vs. G2
*NRP1*	0.5826	0.0003	0.0001	G0 vs. G2
*IGFBP6*	−0.7334	0.0007	0.0004	G0 vs. G2
*IRS1*	−0.4930	0.0007	0.0004	G0 vs. G2
***IGF2***	**0.9849**	**0**.**0045**	**0**.**0026**	G0 vs. G2
*HIF1A*	−0.2941	0.0057	0.0033	G0 vs. G2
*VEGFA*	−0.4553	0.0431	0.0296	G0 vs. G2
***IGF2***	**1.9574**	**2.63E-23**	**1.13E-25**	**G1 vs. G2**
*IGFBP3*	0.6200	2.61E-11	6.90E-13	G1 vs. G2
*NRP1*	0.6150	4.31E-11	1.18E-12	G1 vs. G2
*IGFBP5*	0.3610	0.0002	4.10E-05	G1 vs. G2
*IGF2R*	−0.2329	0.0008	0.0002	G1 vs. G2
*IGFBP6*	−0.4501	0.0017	0.0004	G1 vs. G2
*NRP2*	0.3275	0.0017	0.0005	G1 vs. G2
*IGFBP2*	−0.2644	0.0043	0.0013	G1 vs. G2

Gene, Gene symbol; logFC, logarithmic fold change; FDR, False Discovery Rate; Test, contrast. Here, comparisons were developed, including tissue samples (549 cases), comparing G0 = NT samples (52 cases), G1 = Gleason score equal or lower than 7 (292 cases), G2 = Gleason score higher than 7 (205 cases).

#### miRNA Expression Analysis

MiRTarBase (mirTarBase, 2021) is one of the most comprehensively annotated and experimentally validated miRNA–target interaction databases. According to this database, there are 46 miRNAs which may target *IGF2*.

By the search of these miRNAs, according to Gleason-based differential expression analysis performed on the TCGA-PRAD miRNA database, it was determined that miR-93-5p and miR-200c-3p are those which could have a potential influence on *IGF2* modulation in PC. However, none of them showed any statistical significance in G1 vs. G2. More details can be observed in Table 3.

**TABLE 3:** *IGF2* target miRNAs analysis comparing Gleason scores.

miRNA	logFC	*p* value	FDR	Test
miR-93-5p	1.6018	1.8904e-37	2.5096e-35	G0 vs. G1
miR-200c-3p	1.56318	2.1194e-32	1.2504e-30	G0 vs. G1
miR-100-5p	−0.53748	3.6629e-09	1.5315e-08	G0 vs. G1
miR-320a	0.6363	2.3654e-08	8.5443e-08	G0 vs. G1
let-7a-5p	0.4601	3.5651e-07	1.0942e-06	G0 vs. G1
miR-339-3p	−0.2659	0.0044	0.0076	G0 vs. G1
miR-125b-5p	−0.2182	0.0055	0.0093	G0 vs. G1
miR-200b-3p	0.4704	0.0112	0.0180	G0 vs. G1
miR-320b	0.3298	0.0179	0.0277	G0 vs. G1
miR-93-5p	1.9519	5.3193e-48	9.4151e-46	G0 vs. G2
miR-200c-3p	1.6185	4.8291e-33	1.6027e-31	G0 vs. G2
miR-100-5p	−0.6493	7.6119e-12	2.9940e-11	G0 vs. G2
miR-125b-5p	−0.4584	1.7001e-08	4.8534e-08	G0 vs. G2
let-7a-5p	0.5252	2.0643e-08	5.8619e-08	G0 vs. G2
miR-320a	0.5621	1.2643e-06	2.9706e-06	G0 vs. G2
miR-3200-3p	0.6489	0.0004	0.0007	G0 vs. G2
miR-200b-3p	0.5366	0.0052	0.0079	G0 vs. G2
miR-429	0.5581	0.012	0.0178	G0 vs. G2
miR-320b	0.3375	0.0187	0.0261	G0 vs. G2
miR-150-5p	0.3765	0.0305	0.0413	G0 vs. G2
miR-93-5p	0.3502	2.2858e-08	3.0344e-07	G1 vs. G2
miR-125b-5p	−0.2401	1.2726e-06	1.1855e-05	G1 vs. G2
miR-3200-3p	0.3295	0.0012	0.0045	G1 vs. G2
miR-339-3p	0.1654	0.0044	0.0143	G1 vs. G2

miRNA, miRNA symbol; logFC, logarithmic fold change; FDR, False Discovery Rate; Test, contrast. Here, comparisons were developed, including tissue samples (531 cases), comparing G0 = NT samples (51 cases), G1 = Gleason score equal or lower than 7 (285 cases), G2 = Gleason score higher than 7 (195 cases).

#### TCGA Somatic Mutations

According to the *in silico* analysis and TCGA comparisons, we have obtained several remarkable data in the interactions of *IGF2* with other pathogenic effect variants. That is the case of rs1114167321, rs553443857, rs1057518115, rs1064794050, and rs869320620. When conducting somatic analysis, rs758164144 is the most frequent variant in G1 cluster (Gleason scores ≤7) and, even with low presence, rs3842753 is only present in Gleason scores >7. See more details in Table 4. rs1004446 has also located as a somatic mutation in G1 clustering, data not shown.

**TABLE 4:** Summary of the main somatic mutations in *IGF2* (TCGA-PRAD cohort).

Mut position	Mut id	Total	G1 Gleason score ≤7	G2 Gleason score >7
2133567	rs758164144	7	6	1
2136949	rs3213216	4	2	2
2159830	rs3842753	2	0	2
2160994	rs689	2	2	0

### Functional Analysis

**FIGURE 2:**
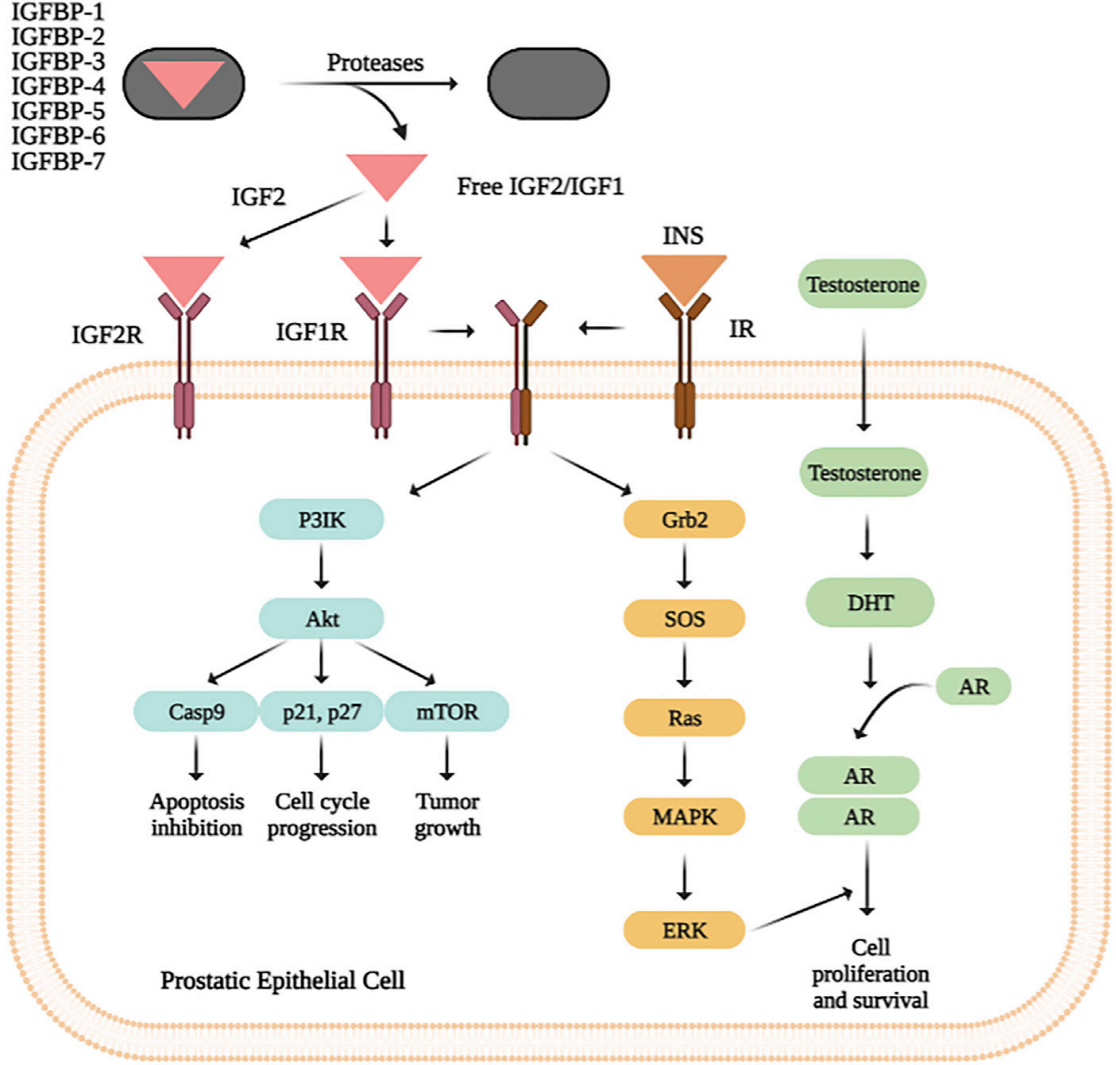
*IGF2*, *IGF1* and insulin bind their specific receptors, which include *IGF1R, IGF2R, IR,* and hybrid receptors. Ligand binding results in autophosphorylation of the tyrosine residues of each receptor, leading to recruitment of the adaptor proteins *IRS* and *Shc* to the intracellular domains of the receptor’s β-subunits. This process activates different signalling cascades through the *PI3K-AKT* and *RAS/RAF/MEK/ERK/ERK* pathways, resulting in stimulation of translation and cell cycle progression, increased proliferation and growth, and inhibition of apoptosis.

A functional analysis in *IGF2* and its 20-interactors genes was performed *IGF2* using STRING (Szklarczyk et al., 2019) and DAVID (Database for Annotation, Visualization and Integrated Discovery) Bioinformatics Resources v6.8 (DAVID, 2020), to obtain the role of gene pool, clinical implication, ontology and involved metabolic pathways. As a result, we found that Proteoglycans in cancer pathway is the most enriched one according to *p*-value (4.6e-08) and FDR (1.6e-06) values. A simple diagram of the *IGF2* pathway can be seen in Figure 2.

### Molecular Analysis

Once the bioinformatic analyses were performed, we tested the obtained results by molecular analysis in blood samples of our population described in Table 1.

#### Linkage Disequilibrium Analysis

For *IGF2* gene, both SNPs (rs1004446 and rs3741211) were linked with a statistic r = 0.9798, therefore we have only analyzed one of them with TaqMan probes (i.e., rs1004446).

#### Association of rs1004446 (*IGF2*) Genotype With Aggressiveness and Treatment Response

In relation to aggressiveness, we compared Gleason scores ≥ or <7; as well as the value of D’Amico risk. None of them showed statistical significance. In the case of treatment response classification, we grouped patients according to sensibility or resistance to treatment, but unfortunately, we could not prove any data with statistic power (Supplementary Table S4).

#### *IGF2* Gene Expression Analysis by qPCR

**FIGURE 3:**
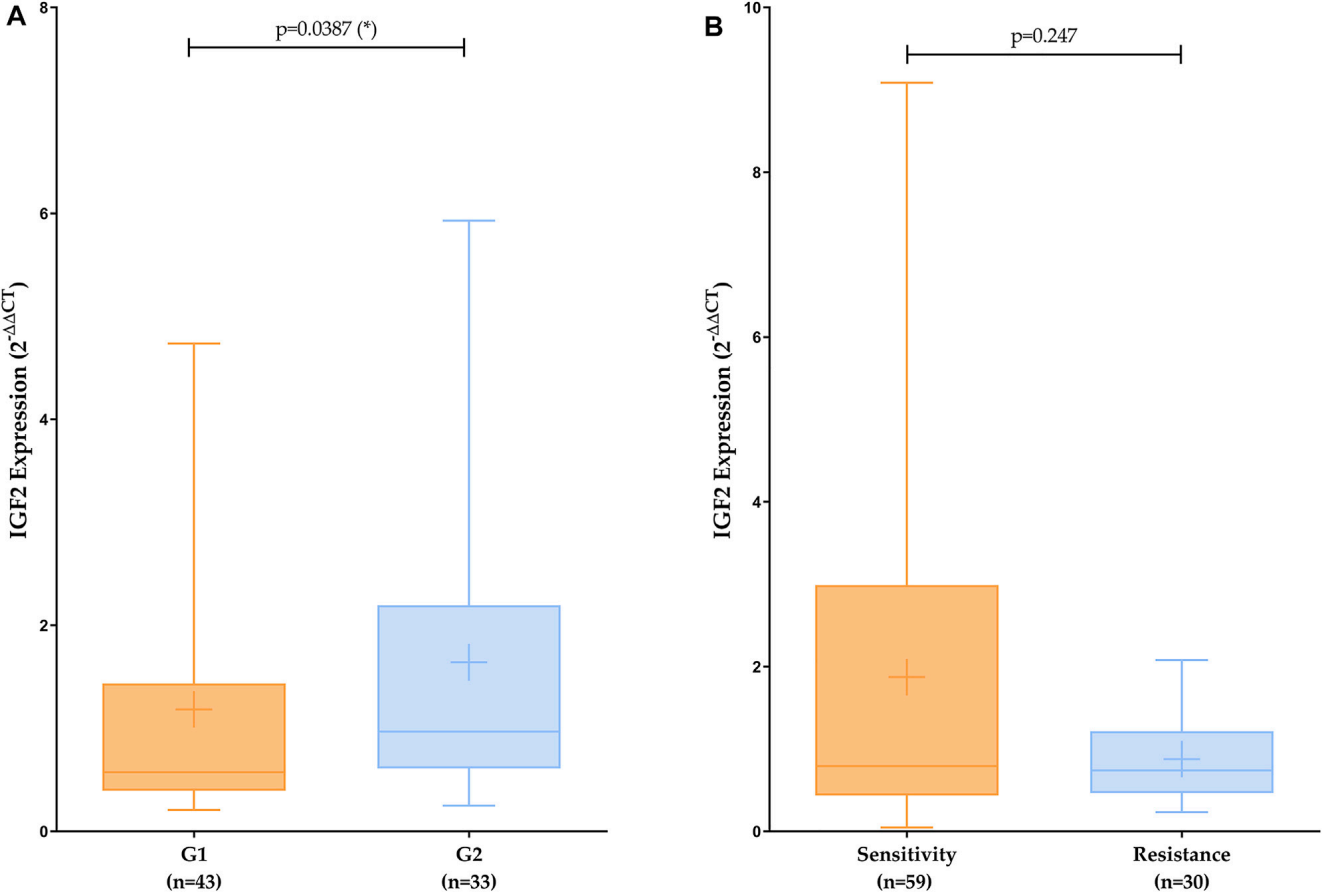
*IGF2* expression analysis comparing aggressiveness **(A)** and treatment response **(B)**. Mean is represented by a plus symbol. 2^−ΔΔCt^ (mean ± SD): G1 = 1.185 ± 1.247; G2 = 1.642 ± 1.542; Sensitivity = 1.8710 ± 2.150; Resistance = 0.8775 ± 0.534.

Results achieved by qPCR of fresh tissue samples were analyzed following ΔΔCt method and using a non-parametric test (Mann Whitney). The level of statistical significance used was *p* < 0.05. The value of genetic expression of each patient was calculated as the average ±SD of three different replicates. A Tukey’s range test was performed to detect anomalous values. To increase the statistical significance and verify our results tendency, analysis was repeated including all replicates from patients as individual values. As can be seen in Figure 3, when comparing aggressiveness, we found similar statistically significant patterns as in the TCGA analysis. Although when analyzing treatment response, we could not observe any significant differences, we can see the same patterns that those observed in bioinformatic analysis.

#### miRNAs Analysis

**FIGURE 4:**
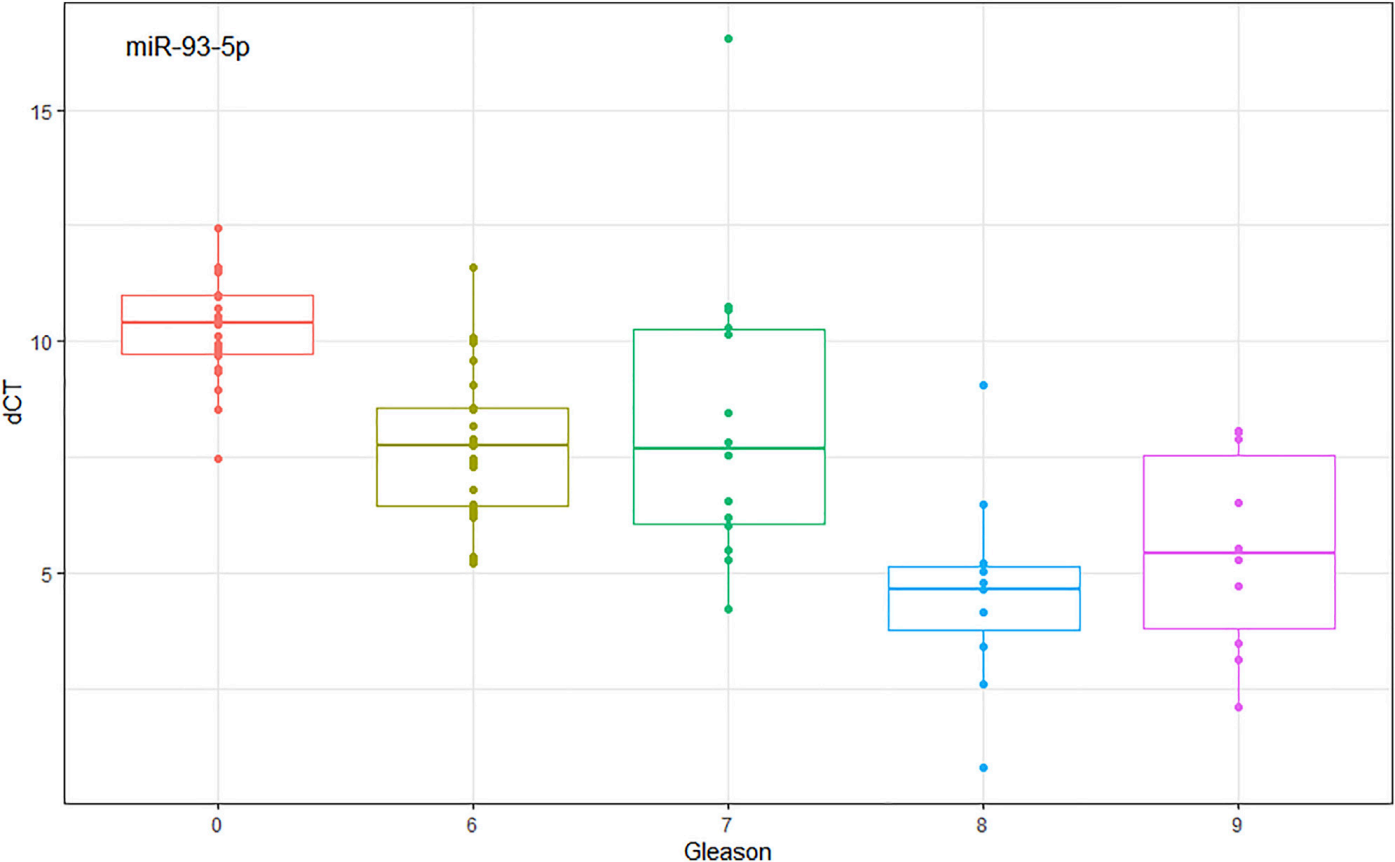
miR-93-5p expression analysis comparing Gleason score. dCt (mean ± SD): Gleason score 0 (NT controls) = 10.258 ± 1.129; Gleason score 6 = 7.692 ± 1.643; Gleason score 7 = 8.280 ± 3.209; Gleason score 8 = 4.622 ± 2.096; Gleason score 9 = 5.477 ± 2.156.

We found by experimental analysis in plasma and tissue samples, that when comparing G1 vs. G2, miR-93-5p is over-expressed according to aggressiveness (Gleason score), the same patterns are repeated with TCGA data. In Figure 4, we can see how miR-93-5p expression changes according to Gleason score. Furthermore, we found that miR-93-5p follows the same expression patterns in both plasma and tissue samples.

## Discussion

Here, we focus on the aim of reinforcing the interesting role of bioinformatic analysis using TCGA database for searching genetic markers in PC. First of all, according to our bioinformatic analysis in PRAD (TCGA), *IGF2* is denoted as one of the most expressed gene in prostate tissue samples. The physiological roles of *IGF2*, as well as its dependence on GH (growth hormone) production, are still controversial. Both *IGF1* and *IGF2* activate a common receptor, the *IGF1* receptor (*IGF1R*), which stimulates mitogenic signals, antiapoptotic and pro-survival activities (Werner et al., 2021). Furthermore, an over-expression of *IGF2BP2* (mRNA binding proteins 2) has been associated with a poor prognosis of the disease in multiple human cancers, as well as, with a shorter survival and poor prognosis in acute myelocytic leukemia, low-grade gliomas, breast , esophageal, hepatocellular, head and neck squamous cell, pancreatic ductal adenocarcinoma and gallbladder carcinomas (Wang et al., 2021). Also, *IGF1R* inhibitors are suggested as anti-cancer drugs because of their effects on proliferation inhibition (Tsui et al., 2021).

Although there are not many data in PC, there are reports about the role of *IGF2*-mRNA and its peptide in PC, with a decrease of 80% in PC compared to non-neoplastic adjacent prostate (Kingshott et al., 2021). There are also data, including the over-expression patterns of IMP3 (messenger RNA binding protein 3 related to *IGF2*) in PC, related to patients’ poor prognosis (Zhang et al., 2020). The results obtained in the present work reveal a significant increase of *IGF2* expression in patients with Gleason scores above 7 in comparation with controls or less aggressive phenotypes of the tumor. Moreover, *IGF2* expression is decreased in treatment resistant PC patients compared with sensitive ones.

Based on our previous results, we searched for the most interesting germline and somatic mutations in *IGF2* related to PC. The combined effect of germline variants, which alter the structure, expression or function of protein-coding regions of cancer-biology related genes; determines which and how many somatic mutations must occur for malignant transformations; that is the reason why we also analyzed them (Qing et al., 2020). Concerning to somatic mutations, we found that rs758164144 is predominantly presented in patients with Gleason scores ≤7 contrasting with rs3842753 clustered in Gleason scores >7. This is the first time that rs758164144 and rs3842753 are described in PC.

Among germline variants, rs1004446 is the top one, according to bioinformatic analysis. This SNP has been previously associated with cancer, such as endometrial cancer risk (McGrath et al., 2011) and PC survival (Cao et al., 2014); or type 1 diabetes (McGrath et al., 2011). However, there are not many details in PC effect. For that reason, we have developed an analysis in blood and buccal swabs samples for testing the effect of this SNP according to aggressiveness or treatment response, but not statistical results were found.

Moreover, when conducting bioinformatic analysis in expression patterns, we discovered that *NRP2* (Neuropilin-2) and *KDR* (Kinase Insert Domain Receptor) genes have the top positions for screening searching (G0 vs. G1 and G0 vs. G2). *NRP2* is a member of the neuropilin receptor family and it is reported to regulate autophagy and *mTORC2* signalling in PC. It has been identified as an important prognostic marker for worse clinical outcome especially in patients with high PC risk (Borkowetz et al., 2020). There is scarce data according to PC but in other tumors such as bladder cancer, high messenger RNA expression of *NRP2, NRP1, PDGFC*, and *PDGFD* are associated with a more aggressive disease (i.e., a high T stage, positive lymph node status and reduced survival) (Förster et al., 2021). Present data reports similarities as previously described in bladder cancer. Concerning *KDR*, there are not many published data *KDR* in PC. Just A. Fraga et al*.* demonstrated that *KDR*−604 T > C was correlated with protein level, accounting for a potential gene-environment effect in the activation of hypoxia-driven pathways in PC (Förster et al., 2021). In colorectal cancer, for example, a significant association was found between *KDR* expression, disease stage and lymph status (Fraga et al., 2017). Here we report higher expression patterns in PC in contrast to controls, as well as differential expression in treatment management.

Finally, we conducted a miRNA analysis, highlighting the role of miR-93-5p and 200c-3p. miR-200c-3p has previously been associated with PC aggressiveness, by its epithelial traits that leads to the anticipation of molecular reprogramming of Zeb1-Slug/vimentin axis (Basu et al., 2020). Recent data also indicated the role in PC progression of miR-200b-3p/200c-3p and *XBP1* (X-box binding protein 1) as critical upstream regulators of *PRKAR2B* (type II-beta regulatory subunit of *PKA*) (Xia et al., 2020). Here we found that miR-200c-3p is situated in the top position according to bioinformatic analysis when comparing Gleason scores classification and PC absence. Thus, this suggest this miRNA as a good screening biomarker option. In relation to miR-93-5p, we have combined bioinformatic analyses with experimental ones, with promising results according to PC aggressiveness and non-invasive biomarkers. miR-93-5p has been previously reported in PC associated with lymphatic dissemination in locally advanced PC (Pudova et al., 2020), or combined with *E2F2* (E2F transcription factor 2), *RRM2* (ribonucleotide reductase regulatory subunit M2), and *PKMYT1* (protein kinase, membrane associated tyrosine/threonine 1) genes and other three miRNAs (hsa-mir-17-5p, hsa-mir-20a-5p, hsa-mir-92a-3p), which marked this miRNA with promising therapeutic options in PC (Wei et al., 2020).

To sum up, here we report the role of *IGF2* as an important marker for aggressiveness in PC. rs1004446 is, for the first time, included as a main somatic and germline mutation in this tumor. Although here, we just found statistically significance when comparing bioinformatic analysis, a deeper analysis with more samples will improve present data. Moreover, *NRP2* and *KDR* have also been included as top screening biomarkers, according to bioinformatic analysis, which opens new strategies in the inclusion of these biomarkers in PC screening. Finally, we found that miR-93-5p could be an efficient strategy as an aggressiveness biomarker with non-invasive techniques.

## Data Availability

Publicly available datasets were analyzed in this study, this data can be found in the Supplementary Material. Further inquiries can be directed to the corresponding author.

## Supplementary Material

Supplementary Figure S1 Representation of samples collection in present study.

Supplementary Table S1 Analysis of *IGF2* Variant effect using VEP of Ensembl and ClinVar database.

Supplementary Table S2 Data from *IGF2* genotyping probe.

Supplementary Table S3 Differential Expression of the *IGF2* gene and IGF2 interacting protein genes using Androgen Deprivation therapy response as factor.

Supplementary Table S4 Association of rs1004446 (*IGF2*) genotype with aggressiveness and treatment response.

## References

[B1] Basu S. Chaudhary A. Chowdhury P. Karmakar D. Basu K. Karmakar D. (2020). Evaluating the Role of Hsa-miR-200c in Reversing the Epithelial to Mesenchymal Transition in Prostate Cancer. Gene 730, 144264. 10.1016/j.gene.2019.144264 31759982

[B2] Benjamini Y. Drai D. Elmer G. Kafkafi N. Golani I. (2001). Controlling the False Discovery Rate in Behavior Genetics Research. Behav. Brain ResearchBehavioural Brain Res. 125, 279–284. 10.1016/s0166-4328(01)00297-2 11682119

[B3] Borkowetz A. Froehner M. Rauner M. Conrad S. Erdmann K. Mayr T. (2020). Neuropilin‐2 Is an Independent Prognostic Factor for Shorter Cancer‐specific Survival in Patients with Acinar Adenocarcinoma of the Prostate. Int. J. Cancer 146, 2619–2627. 10.1002/ijc.32679 31509606

[B4] Broad Institue (2021). Broad GDAC Firehose. Available at: https://gdac.broadinstitute.org/ .

[B15] Cancer Genome Atlas (2021). The Cancer Genome Atlas. Available at: https://www.genome.gov/Funded-Programs-Projects/Cancer-Genome-Atlas .

[B5] Cao Y. Lindström S. Schumacher F. Stevens V. L. Albanes D. Berndt S. (2014). Insulin-like Growth Factor Pathway Genetic Polymorphisms, Circulating IGF1 and IGFBP3, and Prostate Cancer Survival. JNCI J. Natl. Cancer Inst. 106, dju085. 10.1093/jnci/dju085 24824313 PMC4081624

[B6] Chen Q. Sun T. Wang F. Gong B. Xie W. Ma M. (2019). Long Noncoding RNA IGF2AS Is Acting as an Epigenetic Tumor Suppressor in Human Prostate Cancer. Urology 124, 310–e8. 10.1016/j.urology.2018.11.002 30423304

[B7] Creighton C. J. (20182018). Making Use of Cancer Genomic Databases. Curr. Protoc. Mol. Biol. 121, 19.14.1–19.14.13. 10.1002/cpmb.49 PMC5774229 29337373

[B8] DAVID (2020). Functional Annotation Bioinformatics Microarray Analysis. Available at: https://david.ncifcrf.gov/ .

[B9] Ensembl (2021). Ensembl Genome Browser 104. Available at: https://www.ensembl.org/index.html .

[B10] Förster S. Givehchi M. Nitschke K. Mayr T. Kilian K. Dutta S. (2021). Neuropilin-2 and its Transcript Variants Correlate with Clinical Outcome in Bladder Cancer. Genes (Basel). 12. 10.3390/genes12040550 PMC8070368 33918816

[B11] Fraga A. Ribeiro R. Coelho A. Vizcaíno J. R. Coutinho H. Lopes J. M. (2017). Genetic Polymorphisms in Key Hypoxia-Regulated Downstream Molecules and Phenotypic Correlation in Prostate Cancer. BMC Urol. 17, 12. 10.1186/s12894-017-0201-y 28143503 PMC5282787

[B12] Freeman B. Smith N. Curtis C. Huckett L. Mill J. Craig I. W. (2003). DNA from Buccal Swabs Recruited by Mail: Evaluation of Storage Effects on Long-Term Stability and Suitability for Multiplex Polymerase Chain Reaction Genotyping. Behav. Genet. 33, 67–72. 10.1023/a:1021055617738 12645823

[B13] GeneCard (2021). IGF2 Gene - GeneCards | IGF2 Protein | IGF2 Antibody. Available at: https://www.genecards.org/cgi-bin/carddisp.pl?gene=IGF2&keywords=IGF2 .

[B16] Georgiesh T. Namløs H. M. Sharma N. Lorenz S. Myklebost O. Bjerkehagen B. (2021). Clinical and Molecular Implications of NAB2-STAT6 Fusion Variants in Solitary Fibrous Tumour. Pathology 53, 713–719. 10.1016/j.pathol.2020.11.010 33745702

[B17] Gómez-Martín A. Hernández A. F. Martínez-González L. J. González-Alzaga B. Rodríguez-Barranco M. López-Flores I. (2015). Polymorphisms of Pesticide-Metabolizing Genes in Children Living in Intensive Farming Communities. Chemosphere 139, 534–540. 10.1016/j.chemosphere.2015.07.079 26318115

[B18] Harun-Or-Roshid M. Ali M. B. Jesmin, Mollah M. N. H. (2021). Statistical Meta-Analysis to Investigate the Association between the Interleukin-6 (IL-6) Gene Polymorphisms and Cancer Risk. PLoS One 16. 10.1371/journal.pone.0247055 PMC7939379 33684135

[B19] He P. Zhang Z. Huang G. Wang H. Xu D. Liao W. (2016). miR-141 Modulates Osteoblastic Cell Proliferation by Regulating the Target Gene of lncRNA H19 and lncRNA H19-Derived miR-675. Am. J. Transl. Res. 8, 1780–1788. 27186302 PMC4859907

[B14] Kent W. J. (2002). The Human Genome Browser at UCSC. Genome Res. 12 (6), 996–1006. 12045153 10.1101/gr.229102 PMC186604

[B20] Kingshott G. Biernacka K. Sewell A. Gwiti P. Barker R. Zielinska H. (2021). Alteration of Metabolic Conditions Impacts the Regulation of Igf-Ii/h19 Imprinting Status in Prostate Cancer. Cancers (Basel) 13, 1–18. 10.3390/cancers13040825 PMC7920081 33669311

[B27] Landrum M. J. (2018). ClinVar: Improving Access to Variant Interpretations and Supporting Evidence. Nucleic Acids Res.. 10.1093/nar/gkx1153 PMC5753237 29165669

[B21] Makimoto G. Ninomiya K. Kubo T. Sunami R. Kato Y. Ichihara E. (2021). A Novel Osimertinib-Resistant Human Lung Adenocarcinoma Cell Line Harbouring Mutant EGFR and Activated IGF1R. Jpn. J. Clin. Oncol. 51, 956–965. 10.1093/jjco/hyab048 33829270

[B22] Martinez-Gonzalez L. J. Pascual Geler M. Robles Fernandez I. Cozar J. M. Lorente J. A. Alvarez Cubero M. J. (2018). Improving the Genetic Signature of Prostate Cancer, the Somatic Mutations. Urol. Oncol. 36, 312–e23. 10.1016/j.urolonc.2018.03.012 29650325

[B23] McCarthy D. J. Chen Y. Smyth G. K. (2012). Differential Expression Analysis of Multifactor RNA-Seq Experiments with Respect to Biological Variation. Nucleic Acids Res. 40, 4288–4297. 10.1093/nar/gks042 22287627 PMC3378882

[B24] McGrath M. Lee I.-M. Buring J. De Vivo I. (2011). Common Genetic Variation within IGFI, IGFII, IGFBP-1, and IGFBP-3 and Endometrial Cancer Risk. Gynecol. Oncol. 120, 174–178. 10.1016/j.ygyno.2010.10.012 21078522 PMC3238452

[B25] McLaren W. Gil L. Hunt S. E. Riat H. S. Ritchie G. R. S. Thormann A. (2016). The Ensembl Variant Effect Predictor. Genome Biol. 17, 122. 10.1186/s13059-016-0974-4 27268795 PMC4893825

[B26] miRTarBase (2021). MIRTARBase. Available at: https://mirtarbase.cuhk.edu.cn/ .

[B28] NCBI (2019). Medicine. Bethesda, Maryland: N. L. of. National Center for Biotechnology Information.

[B29] NIH (2021a). GDC. Available at: https://portal.gdc.cancer.gov/ .

[B32] Pearce C. L. Doherty J. A. Van Den Berg D. J. Moysich K. Hsu C. Cushing-Haugen K. L. (2011). Genetic Variation in Insulin-like Growth Factor 2 May Play a Role in Ovarian Cancer Risk. Hum. Mol. Genet. 20, 2263–2272. 10.1093/hmg/ddr087 21422097 PMC3090188

[B33] Pudova E. A. Krasnov G. S. Nyushko K. M. Kobelyatskaya A. A. Savvateeva M. V. Poloznikov A. A. (2020). MiRNAs Expression Signature Potentially Associated with Lymphatic Dissemination in Locally Advanced Prostate Cancer. BMC Med. Genomics 13, 129. 10.1186/s12920-020-00788-9 32948204 PMC7500008

[B34] Qing T. Mohsen H. Marczyk M. Ye Y. O'Meara T. Zhao H. (2020). Germline Variant burden in Cancer Genes Correlates with Age at Diagnosis and Somatic Mutation burden. Nat. Commun. 11, 2438–8. 10.1038/s41467-020-16293-7 32415133 PMC7228928

[B35] Robinson M. D. Oshlack A. (2010). A Scaling Normalization Method for Differential Expression Analysis of RNA-Seq Data. Genome Biol. 11, R25. 10.1186/gb-2010-11-3-r25 20196867 PMC2864565

[B36] Robinson M. D. McCarthy D. J. Smyth G. K. (2009). edgeR: a Bioconductor Package for Differential Expression Analysis of Digital Gene Expression Data. Bioinformatics 26, 139–140. 10.1093/bioinformatics/btp616 19910308 PMC2796818

[B37] Saunders E. J. Kote‐jarai Z. Eeles R. A. (2021). Identification of Germline Genetic Variants that Increase Prostate Cancer Risk and Influence Development of Aggressive Disease. Cancers 13, 1–26. 10.3390/cancers13040760 PMC7917798 33673083

[B40] Szklarczyk D. Gable A. L. Lyon D. Junge A. Wyder S. Huerta-Cepas J. (2019). STRING v11: Protein-Protein Association Networks With Increased Coverage, Supporting Functional Discovery in Genome-Wide Experimental Datasets. Nucleic Acids Res. 47 (D1), D607–D613. 30476243 10.1093/nar/gky1131 PMC6323986

[B38] Seol H. S. Akiyama Y. Lee S. E. Shimada S. Jang S. J. (2020). Loss of miR-100 and miR-125b Results in Cancer Stem Cell Properties through IGF2 Upregulation in Hepatocellular Carcinoma. Sci. Rep. 10, 21412–21511. 10.1038/s41598-020-77960-9 33293585 PMC7722933

[B39] Solé X. Guinó E. Valls J. Iniesta R. Moreno V. (2006). SNPStats: a Web Tool for the Analysis of Association Studies. Bioinformatics 22, 1928–1929. 10.1093/bioinformatics/btl268 16720584

[B41] Tsui J. Qi S. Perrino S. Leibovitch M. Brodt P. (2021). Identification of a Resistance Mechanism to Igf‐ir Targeting in Human Triple Negative Mda‐mb‐231 Breast Cancer Cells. Biomolecules 11. 10.3390/biom11040527 PMC8065809 33916323

[B42] Wang C. Li J. (2021). A Deep Learning Framework Identifies Pathogenic Noncoding Somatic Mutations from Personal Prostate Cancer Genomes. Cancer Res. 80, 4644–4654. 10.1158/0008-5472.CAN-20-1791 32907840

[B43] Wang J. Chen L. Qiang P. (2021). The Role of IGF2BP2, an m6A Reader Gene, in Human Metabolic Diseases and Cancers. Cancer Cel Int. 21, 99. 10.1186/s12935-021-01799-x PMC7876817 33568150

[B45] Wei J. Yin Y. Deng Q. Zhou J. Wang Y. Yin G. (2020). Integrative Analysis of MicroRNA and Gene Interactions for Revealing Candidate Signatures in Prostate Cancer. Front. Genet. 11, 176. 10.3389/fgene.2020.00176 32180804 PMC7057858

[B46] Weiner A. B. Faisal F. A. Davicioni E. Karnes R. J. Griend D. J. V. Lotan T. L. (2020). Somatic HOXB13 Expression Correlates with Metastatic Progression in Men with Localized Prostate Cancer Following Radical Prostatectomy. Eur. Urol. Oncol., 30055–30059. 10.1016/j.euo.2020.05.001 PMC7736205 32540218

[B47] Werner H. Sarfstein R. Laron Z. (2021). The Role of Nuclear Insulin and Igf1 Receptors in Metabolism and Cancer. Biomolecules 11, 531. 10.3390/biom11040531 33918477 PMC8065599

[B48] WHO (2020). Cancer Today. Available at: https://gco.iarc.fr/today/online-analysis-table?v=2020&mode=cancer&mode_population=continents&population=900&populations=900&key=asr&sex=0&cancer=39&type=0&statistic=5&prevalence=0&population_group=0&ages_group%5B%5D=0&ages_group%5B%5D=17&group_cancer=1&include_nmsc=1&include_nmsc_other=1 .

[B49] Xia L. Han Q. Chi C. Zhu Y. Pan J. Dong B. (2020). Transcriptional Regulation of PRKAR2B by miR-200b-3p/200c-3p and XBP1 in Human Prostate Cancer. Biomed. Pharmacother. 124, 109863. 10.1016/j.biopha.2020.109863 31986411

[B50] Xing S. Tian Z. Zheng W. Yang W. Du N. Gu Y. (2021). Hypoxia Downregulated miR-4521 Suppresses Gastric Carcinoma Progression through Regulation of IGF2 and FOXM1. Mol. Cancer 20, 9. 10.1186/s12943-020-01295-2 33407516 PMC7786912

[B51] Xu X. Yu Y. Zong K. Lv P. Gu Y. (2019). Up-regulation of IGF2BP2 by Multiple Mechanisms in Pancreatic Cancer Promotes Cancer Proliferation by Activating the PI3K/Akt Signaling Pathway. J. Exp. Clin. Cancer Res. 38, 497. 10.1186/s13046-019-1470-y 31852504 PMC6921559

[B31] Ye J. Coulouris G. Zaretskaya I. Cutcutache I. Rozen S. Madden T. L. (2012). Primer-BLAST: A Tool to Design Target-Specific Primers for Polymerase Chain Reaction. mBMC Bioinformatics 13, 134. 10.1186/1471-2105-13-134 PMC3412702 22708584

[B52] Yun J. W. Yang L. Park H. Y. Lee C. W. Cha H. Shin H. T. (2020). Dysregulation of Cancer Genes by Recurrent Intergenic Fusions. Genome Biol. 21, 166. 10.1186/s13059-020-02076-2 32631391 PMC7339451

[B53] Zhang X. Wang D. Liu B. Jin X. Wang X. Pan J. (2020). IMP3 Accelerates the Progression of Prostate Cancer through Inhibiting PTEN Expression in a SMURF1-dependent Way. J. Exp. Clin. Cancer Res. 39, 190. 10.1186/s13046-020-01657-0 32938489 PMC7493339

