# Supplementary material for: Role of IGF2 in the Study of Development and Evolution of Prostate Cancer
Source: Front Genet. 2022 Jan 4;12:740641. doi: 10.3389/fgene.2021.740641 (PMC8790605; doi:10.3389/fgene.2021.740641)
Supplement: Supplementary file 1 [file DataSheet2.docx]

**Role of IGF2 in the study of development and evolution of prostate cancer.**

P. Porras-Quesada^1†^, JM. González-Cabezuelo^2†*^, V. Sánchez-Conde^3^, I. Puche-Sanz^3^, V. Arenas-Rodríguez^1^, C. García-López^4^, JF. Flores-Martín^5^, JM. Molina-Hernández^6^, MJ. Álvarez-Cubero^1,7,8^, LJ. Martínez-González^1^, F. Vázquez-Alonso^3^.

Publicly available datasets were analyzed in this study. This data can be found here:

TCGA-PRAD mRNA-Seq data:

<http://gdac.broadinstitute.org/runs/stddata__2016_01_28/data/PRAD/20160128/>

folder: gdac.broadinstitute.org_PRAD.mRNAseq_Preprocess.Level_3.2016012800.0.0.tar.gz

TCGA-PRAD miRNA-Seq data (Isoform Expression Quantification files):

<https://portal.gdc.cancer.gov/repository?facetTab=files&filters=%7B%22op%22%3A%22and%22%2C%22content%22%3A%5B%7B%22content%22%3A%7B%22field%22%3A%22cases.project.project_id%22%2C%22value%22%3A%5B%22TCGA-PRAD%22%5D%7D%2C%22op%22%3A%22in%22%7D%2C%7B%22op%22%3A%22in%22%2C%22content%22%3A%7B%22field%22%3A%22files.data_type%22%2C%22value%22%3A%5B%22Isoform%20Expression%20Quantification%22%5D%7D%7D%2C%7B%22content%22%3A%7B%22field%22%3A%22files.experimental_strategy%22%2C%22value%22%3A%5B%22miRNA-Seq%22%5D%7D%2C%22op%22%3A%22in%22%7D%5D%7D&searchTableTab=files>

TCGA-PRAD clinical data:

<https://portal.gdc.cancer.gov/projects/TCGA-PRAD>

TCGA-PRAD annotated somatic mutations (MuTect2) files:

https://portal.gdc.cancer.gov/repository?facetTab=files&filters=%7B%22op%22%3A%22and%22%2C%22content%22%3A%5B%7B%22content%22%3A%7B%22field%22%3A%22cases.project.project_id%22%2C%22value%22%3A%5B%22TCGA-PRAD%22%5D%7D%2C%22op%22%3A%22in%22%7D%2C%7B%22op%22%3A%22in%22%2C%22content%22%3A%7B%22field%22%3A%22files.analysis.workflow_type%22%2C%22value%22%3A%5B%22MuTect2%20Annotation%22%5D%7D%7D%2C%7B%22op%22%3A%22in%22%2C%22content%22%3A%7B%22field%22%3A%22files.data_category%22%2C%22value%22%3A%5B%22Copy%20Number%20Variation%22%2C%22copy%20number%20variation%22%2C%22simple%20nucleotide%20variation%22%5D%7D%7D%2C%7B%22op%22%3A%22in%22%2C%22content%22%3A%7B%22field%22%3A%22files.data_type%22%2C%22value%22%3A%5B%22Annotated%20Somatic%20Mutation%22%5D%7D%7D%5D%7D&searchTableTab=files
