# Supplementary material for: Role of IGF2 in the Study of Development and Evolution of Prostate Cancer
Source: Front Genet. 2022 Jan 4;12:740641. doi: 10.3389/fgene.2021.740641 (PMC8790605; doi:10.3389/fgene.2021.740641)
Supplement: Supplementary file 2 [file DataSheet1.docx]

**Supplementary Material:**

**
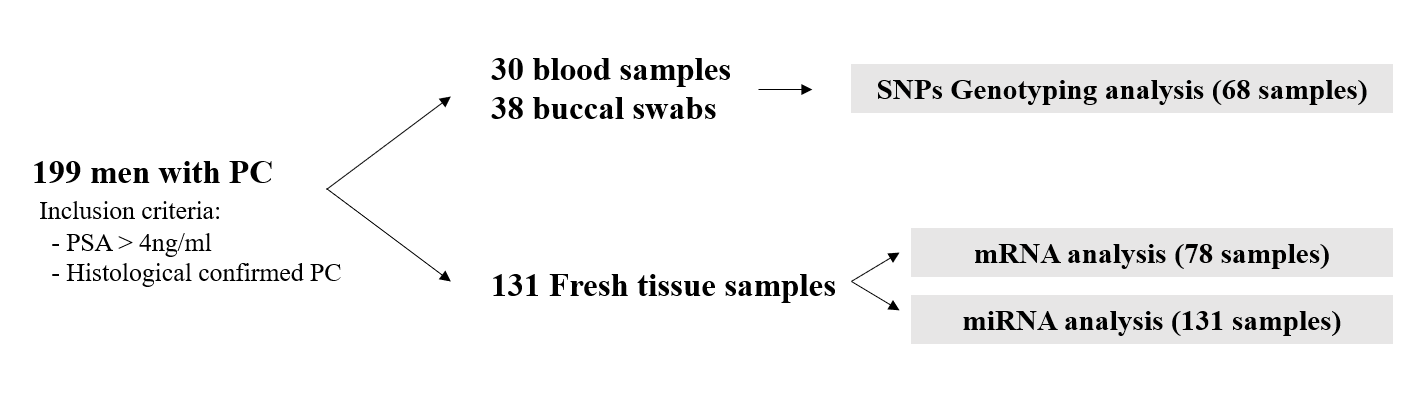
Supplementary Figure 1. Representation of samples collection in present study.**

**Supplementary Table 1. Analysis of *IGF2* Variant effect using VEP of Ensembl and ClinVar database.**

| **Variant Effect** | **Number of Variants (%)** |
| --- | --- |
| Coding Variant  Intronic Variant  UTR Variant  3’UTR Variant  3’ Downstream Variant  Non-synonymous Variant  5’ Upstream Varian  Synonymous Variant. | 26.50  18.62  15.49  15.13  10.83  6.45  5.55  1.43 |
| **SNPs** | **Clinical** **Significance** |
| rs1114167321  rs553443857  rs1057518115  rs1064794050  rs869320620  rs755455183  rs748459239  rs1564894932 | Pathogenic  Pathogenic  Likely pathogenic  Pathogenic  Pathogenic  Uncertain  Uncertain  Uncertain |

**Supplementary Table 2. Data from *IGF2* genotyping probe.**

| **SNP Genotyping, Taq-Man Assay (ThermoFisher)** |
| --- |
| [**C___1223303_10**](https://www.thermofisher.com/order/genome-database/details/genotyping/C___1223303_10?CID=&ICID=&subtype=)  Catalog number: [**4351379**](https://www.thermofisher.com/order/catalog/product/4351379)  **SNP ID:**  [rs1004446](http://www.ncbi.nlm.nih.gov/SNP/snp_ref.cgi?searchType=adhoc_search&type=rs&rs=rs1004446)  **Gene:**  *IGF2*  **Gene Name:**  insulin like growth factor 2  **Location**:  Chr.11: 2148913 on GRCh38  **Polymorphism:**  A/G, Transition Substitution  **Context Sequence [VIC/FAM]:**  TCAGTGGCTTTGTGCAGCCCTAAAG**[A/G]**AGAACCCTCTCCACAATGGCCTTTC |

**Supplementary Table 3. Differential Expression of the *IGF2* gene and IGF2 interacting protein genes using Androgen Deprivation therapy response as factor.**

| **Gene** | **LogFC** | **FDR** | **PValue** | **Test** |
| --- | --- | --- | --- | --- |
| ***IGF2*** | -3,7897 | 0.0356 | 3.34E-05 | DR1 vs. DR2 |
| *HIF1A* | -0.3258 | 0.9993 | 0.0750 | DR1 vs. DR2 |
| *INSR* | -0.1866 | 0.9993 | 0.1172 | DR1 vs. DR2 |
| *IGF1* | -0.7810 | 0.9993 | 0.1578 | DR1 vs. DR2 |
| *NRP1* | -0.3453 | 0.9993 | 0.2148 | DR1 vs. DR2 |
| *KDR* | -1.5433 | 0.4511 | 0.0021 | DR1 vs. DR3 |
| ***IGF2*** | -5.9003 | 0.4989 | 0.0061 | DR1 vs. DR3 |
| *NRP2* | -1.7592 | 0.5063 | 0.0107 | DR1 vs. DR3 |
| *FLT1* | -1.2236 | 0.5312 | 0.0344 | DR1 vs. DR3 |
| *IGFBP4* | -1.1746 | 0.5312 | 0.0370 | DR1 vs. DR3 |
| *KDR* | -1.7319 | 0.3569 | 0.0009 | DR2 vs. DR3 |
| *HIF1A* | 0.9049 | 0.4231 | 0.0068 | DR2 vs. DR3 |
| *NRP2* | -1.4243 | 0.5395 | 0.0359 | DR2 vs. DR3 |
| *GPC3* | -1.2143 | 0.5527 | 0.0464 | DR2 vs. DR3 |
| *IRS1* | -1.0765 | 0.5732 | 0.0664 | DR2 vs. DR3 |

Footnote Table ST3. Gene (Gene symbol); logFC (logarithmic fold change); FDR (False Discovery Rate); Test (contrast). Here, comparisons were developed, including tissue samples (73 cases), comparing DR1: treatment based on a single drug target (43 cases), DR2: treatment in which a new drug target has been prescribed due to failure of the first one (25 cases), DR3: chemotherapy (5 cases).

**Supplementary Table 4. Association of rs1004446 (*IGF2*) genotype with aggressiveness and treatment response.**

| **Variables** | **G/G, n(%)** | **A/A-A/G, n(%)** | **p-value^a^** | **OR** | **95% CI** |
| --- | --- | --- | --- | --- | --- |
| Treatment Response (n=65)*  Sensitivity (n=18)  Resistance (n=47) | 9 (31.03%)  20 (68.97%) | 9 (25%)  27 (75%) | 0.589 | 1.350 | 0.454-4.015 |
| Gleason score (n=56)*  ≤7 (n=28)  >7 (n=28) | 12 (46.15%)  14 (53.85%) | 16 (51.61%)  15 (48.39%) | 0.682 | 0.804 | 0.283-2.283 |
| D’Amico Risk Classification (n=49)*  Low / Medium (n=16)  High (n=33) | 6 (27.27%)  16 (72.73%) | 10 (37.04%)  17 (62.96%) | 0.468 | 0.638 | 0.188-2.161 |

Footnote Table ST4. *Missing clinical data makes differences in samples´ collection in each group.

^a^ Pearson’s chi-squared test (ꭓ^2^);
